# Recent advances in the understanding and management of erectile dysfunction

**DOI:** 10.12688/f1000research.16576.1

**Published:** 2019-01-25

**Authors:** Sarah C Krzastek, Justin Bopp, Ryan P Smith, Jason R Kovac

**Affiliations:** 1 Department of Urology, University of Virginia, Charlottesville, Virginia, USA; 2 Men's Health Center, Indianapolis, Indiana, USA

**Keywords:** medical therapy, penile injections, belted prosthesis, external penile prosthesis

## Abstract

Erectile dysfunction (ED) is important to a man’s well-being and health, since it not only affects the individual but also causes strain on a couple’s lifestyle and relationship. There are multiple non-invasive treatments that exist for ED including lifestyle changes, oral medications (phosphodiesterase type 5 inhibitors), vacuum-assisted erectile devices, and intraurethral suppositories. While lifestyle changes and oral medications are typically first-line treatments for ED, more-invasive treatments including intracavernosal injections and surgically implanted prosthetic devices may be required for the management of complex cases. Additionally, novel therapies are currently being developed, and future treatment options may include shock-wave therapy, external prosthetic devices, and injection of stem cells or platelet-rich plasma. The current manuscript seeks to highlight advances in management and may eventually alter the treatment paradigm to allow more-inclusive care pathways.

## Introduction

Erectile dysfunction (ED), one of the most frequently reported medical conditions in men, is defined as the chronic inability to achieve or sustain a penile erection. Indeed, regular and chronic ED increases with age from ~35% of men aged 60 to ~50% of men older than 70 being affected ^1^. Primary care physicians identify and diagnose the majority of ED in the United States and Canada and, as such, are on the ground floor of helping patients navigate the multitude of treatment options that exist ^2^.

Many risk factors for ED exist including age, coronary artery disease, obesity, smoking, depression, hypertension, prior pelvic surgery, and spinal cord injuries as well as other psychological variables ^3^. Sexual health and erectile quality are key components to not only an individual’s quality of life but also their partner’s mental, emotional, and physical well-being ^4^. Along those lines, many seek support as they navigate their way through the multiple treatment options. As erectile function is a complex interplay between psychological and physiological factors, the American Urological Association (AUA) guidelines suggest referral to a mental health professional as adjunctive therapy for the treatment of ED ^5^. This has been shown to improve adherence to treatment plans and improve erectile function when used as a primary therapy and may enhance the effects of additional treatment options ^6^. A partner’s willingness to try different options has been shown to increase the likelihood of recovery of sexual function ^7^. These treatments vary from non-invasive to invasive. While non-invasive treatment options may be preferred for many patients, all management options should be thoroughly discussed. The AUA guidelines acknowledge that any treatment option may be used as a first-line therapy ^5^. Treatment options include lifestyle changes, oral medications, penile injections, and surgically implantable penile prostheses as well as more novel approaches such as belted external penile prostheses. Additional novel approaches to the management of ED are still considered experimental and include penile shockwave therapy and the injection of stem cells or platelet-rich plasma (PRP). These therapies have shown promising initial results and may become part of the ED treatment algorithm in the future. The current manuscript details these treatments with the goal being to help patients understand their options and thus improve their overall sexual recovery and experiences.

## Treatment options

### Lifestyle changes

Improvements in lifestyle and the proper management of medical comorbidities are arguably the most beneficial and safest treatment. This first-line approach is, without a doubt, the most difficult to execute. Patient education detailing the risk factors for ED is a logical first step. While the risks for developing future ED are sometimes out of an individual’s control, understanding them can help to change current behaviors. Lack of physical activity, obesity, unhealthy diets, and cigarette smoking have all been shown to contribute to ED ^8^. Other contributing factors include diabetes, cardiovascular disease, hypertension, hyperlipidemia, metabolic syndrome, and hypogonadism as well as psychiatric and psychological disorders ^8^. Lifestyle changes can thus prevent progression or improve regression in early manifestations of ED ^8^.

Data have found that physical activity is associated with a lower risk of ED while simultaneously both preventing and improving ED ^9^. In a large study of 31,724 men who were free of ED at baseline, a 40% increased risk of ED was noted with the development of obesity ^10^. Weight loss was also beneficial on quality of sexual life ^11^ while a healthy diet and reduced caloric intake have been associated with improvements in erectile function ^8^. Smoking has a negative relationship with erectile function, with cumulative smoking history being correlated to significantly increased ED risk ^12^.

Aside from altering risk factors, some lifestyle adaptations may also play a role in erectile function management. For example, some couples may serve to eroticize some treatments to make them more successful. Kukula *et al.* ^7^ strategized that by taking an erectile aid and incorporating it into the sexual experience in a positive way, the aid itself could become viewed as erotic and stimulating.

### Oral medications

**Figure 1.**
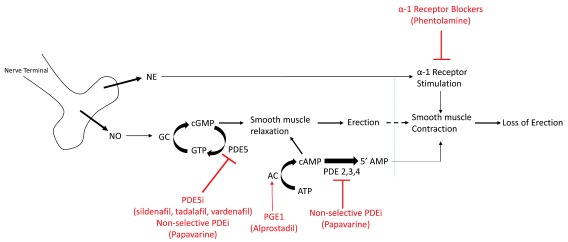
Molecular pathway of erections (black) and location of action of medications for erectile dysfunction (red). AC, adenylyl cyclase; ATP, adenosine triphosphate; cAMP, cyclic adenosine monophosphate; cGMP, cyclic guanosine monophosphate; GC, guanylate cyclase; GTP, guanosine triphosphate; NE, norepinephrine; NO, nitric oxide; PDE5, phosphodiesterase type 5; PDE5i, phosphodiesterase type 5 inhibitor; PDE, phosphodiesterase; PDEi, phosphodiesterase inhibitor; PGE1, prostaglandin E1.

An erection starts with nerve stimulation, which releases nitric oxide (NO). NO stimulates guanylate cyclase (GC), which converts guanosine triphosphate (GTP) into cyclic guanosine monophosphate (cGMP). cGMP subsequently induces smooth muscle relaxation, which allows for influx of blood into the penis with resultant erection. The molecule phosphodiesterase type 5 (PDE5) breaks down cGMP and allows the penis to return to the flaccid state. This is the site of action of the most common oral medications used to treat ED ( Figure 1).

Taken by mouth prior to intercourse, oral medications are the first line of treatment for ED refractory to lifestyle modifications. These medications inhibit PDE5, which keeps the level of cGMP high and promotes erections. The most well-known of these PDE5 inhibitors is sildenafil (Viagra®, Pfizer Inc., FDA approved 1998). Additional PDE5 inhibitors include tadalafil (Cialis®, Lilly, FDA approved 2003), vardenafil (Levitra®, Bayer Healthcare, FDA approved 2003), and avanafil (Stendra®, Metuchen Pharmaceutical, FDA approved 2012).

Sildenafil and vardenafil are similar in action, with a peak absorption of 30–60 minutes and a half-life of 3–5 hours. These medications should be taken on an empty stomach to maximize absorption ^13, 14^. Avanafil is absorbed in 20–30 minutes with a half-life of 6 hours ^15^. Tadalafil is different from the other medications in that it takes longer to absorb (2–4 hours) and has a longer half-life (17.5 hours), so it lasts longer in the body. Additionally, it is not affected by food, so it does not have to be taken on an empty stomach. Currently, tadalafil is the only oral ED medication approved for daily use, rather than on an as-needed basis ^16^. Additionally, it has been shown to improve lower urinary tract symptoms in patients with benign prostatic hyperplasia ^17^.

For these medications to work, the nervous system pathways must be intact and a patient must have some sexual stimulation in order to increase the NO and cGMP in the penile tissues. Therefore, in patients who have severe diabetes with peripheral neuropathy or have undergone radical prostatectomy for prostate cancer, in whom the nerves for erections may be damaged, these medications may not be as effective ^18, 19^. Additionally, oral medications cannot be taken by patients who are taking nitrates for the treatment of chest pain, and care should be taken when combining PDE5 inhibitors with alpha-receptor-blocking medications, which may be prescribed for hypertension and lower urinary tract symptoms ^20^.

Even if a patient has tried oral medications in the past with poor response, he may have a good response with an alternative oral medication ^21^. A recent review of the literature and meta-analysis showed that sildenafil and tadalafil had similar efficacy and side effect profiles, but tadalafil had improved psychological outcomes including satisfaction with ED treatment ^22^, suggesting that this should be considered when deciding which oral medication to prescribe. Some evidence suggests that combining daily tadalafil with on-demand sildenafil may result in improved erectile function, particularly in men with severe ED ^23^. Additionally, for men who have difficulty timing an on-demand PDE5 inhibitor with sexual intercourse or for men experiencing bothersome side effects on higher-dose on-demand medications, the once-daily formulation of tadalafil may allow for better medication compliance, fewer side effects, and therefore better outcomes on the medication ^24^. There has been some recent controversy as to whether these medications can cause melanoma skin cancer or prostate cancer, but there is currently no evidence to support this, and PDE5 inhibitors remain common and recommended first-line treatments for ED ^25^.

Some patients may find PDE5 inhibitors cost-prohibitive for use in treating their ED. Urology offices often work with compounding pharmacies that may be able to provide these medications at a fraction of the cost. Additionally, several of the name-brand PDE5 inhibitors have recently come off, or soon will be coming off, patent, which will allow the generic versions of these medications to be more affordable. Generic sildenafil became available in 2016, and a generic version of tadalafil was FDA approved in May 2018.

### Intracavernosal injections

Intracavernosal injections (ICIs) are an alternative to oral medications in the treatment of ED. With this treatment, medication is injected directly into the penile corpora at the lateral base of the penis, with care taken to avoid the neurovascular bundles dorsally and the urethra ventrally. The most commonly injected medication is prostaglandin E1 (PGE1), which stimulates cyclic adenosine monophosphate (cAMP) to induce smooth muscle relaxation and promote erections ( Figure 1). PGE1, also known as alprostadil (Edex®, Caverject®) is FDA approved for monotherapy injection. This medication is also commonly combined with one or two other medications, papaverine and phentolamine, as “bimix” or “trimix” in variable formulations for injection to treat ED ( Figure 1).

While slightly more invasive, ICI may be preferred to oral medications for the treatment of ED in certain patients in whom oral medications may be contraindicated (for example, patients who have had a recent heart attack) or who have not been able to tolerate oral medications in the past because of side effects. Additionally, since these medications act directly on the molecules that induce erections, they may work better in patients who have damage to the nerves that stimulate erections (see section on oral medications, and Figure 1) ^26^. The main barrier to the use of this therapy is patient anxiety centered around penile injections ^27^. Conflicting evidence exists as to whether patients have higher satisfaction with the use of ICI or oral PDE5i, but it is known that patients who use ICI consistently have high satisfaction rates ^26^.

According to the AUA guidelines, an in-office injection test dose should be administered to all men considering ICI for the management of ED to optimize the dose and to ensure the patient does not develop priapism or systemic side effects ^26^. The use of this medication is contraindicated in patients with a history of recurrent priapism, Peyronie’s disease, and bleeding disorders ^26^.

### Intraurethral suppositories

Similar to alprostadil ICI, alprostadil is also available as an intraurethral suppository. This route of administration may be preferred by some patients who wish to avoid oral or injectable medications. While intraurethral alprostadil improves erectile function over placebo, it is less effective than ICI ^26^.

It is recommended that a test dose of intraurethral alprostadil be administered in clinic, similar to ICI ^26^. The most common side effect is penile or urethral pain ^28^. This medication should be used with caution in patients with increased risk for priapism or in patients with urethral disease. A condom should be used during sexual activity with a pregnant female partner to minimize her potential risk of exposure to prostaglandin.

### Vacuum-assisted erectile devices

The vacuum-assisted erectile device (VED) is a device that is placed over the penis and pumped to create a vacuum, which pulls blood into the penis to cause engorgement and erection. A band is then placed around the base of the penis to maintain the erection and is removed to allow the penis to return to the flaccid state. The device may be challenging to use for patients with decreased dexterity or a large amount of lower abdominal fat and buried penis. VEDs may be effective in generating an erection in 90% of patients, though long-term use decreases, as many patients feel the devices are inconvenient to use and may have pain and temporary changes to penile sensation with the use of the constricting ring ^24^. Caution should be used in patients on blood thinners, given the theoretical increased risk of penile bruising with the device.

Though penile rehabilitation following radical prostatectomy for prostate cancer remains controversial, a VED may be prescribed as part of a rehabilitation program to minimize the risk of corporal fibrosis and has been shown to assist with erectile function during treatments ^29^.

### Penile prostheses

The penile prosthesis is a surgically implanted device that has been used as a treatment for ED since the 1970s ^30^. The device and the technical aspects of surgical implantation have undergone multiple revisions over the years to optimize device function, minimize device failure, and maximize patient and partner satisfaction. Currently, patients who have undergone placement of a penile prosthesis report the highest satisfaction rate compared to patients who have received oral medications or ICI ^31, 32^.

The penile prosthesis comes in a variety of forms, including a malleable or inflatable device. The inflatable devices may be two- or three-piece devices. The malleable device consists of two rigid cylinders that are implanted within the penile corpora. This device remains rigid and may be simply positioned to allow for intercourse. This device is good for patients with limited dexterity. The inflatable devices consist of two fluid-filled cylinders that are implanted within the penis, along with a pump that is placed within the scrotum, and typically a fluid reservoir which is placed in the abdomen. To induce an erection, the patient squeezes the pump within the scrotum, which pulls fluid from the reservoir to inflate the penile cylinders. To return to the flaccid state, the patient pushes a button on the pump and the fluid leaves the cylinders and returns to the reservoir. The inflatable device results in a more physiologic-appearing erection than the malleable prosthesis.

The penile prosthesis is typically reserved for patients who have not responded to less-invasive ED treatments, though it may be considered as a first-line therapy. Preoperative patient counselling is important to manage postoperative outcomes and expectations ^33^.

## Novel therapies

As many patients fail to respond to traditional therapies for ED, research is ongoing into the development of novel treatment approaches. These treatment options are still considered experimental and, per the AUA guidelines and the Sexual Medicine Society of North America Position Statement on Restorative Therapies for ED, should not be administered outside of a research setting in compliance with Institutional Review Board approval ^5, 34^.

### Penile shockwave therapy

Low-intensity extracorporeal shockwave therapy (LI-ESWT) has been investigated as a therapy that may improve blood vessel function and promote the growth of new blood vessels in the setting of cardiovascular disease. Based on this, groups have begun investigating LI-ESWT as a potential treatment option for ED caused by vascular disease ^35^. A protocol for this therapy was first developed by Vardi *et al*. in 2010, and this group was able to show that LI-ESWT for ED is well tolerated and results in significant improvement in penile blood flow, which was correlated with improvement in erectile function six months following treatment ^35^. While still experimental, LI-ESWT has more recently been shown to improve patient response to oral PDE5 inhibitors and may allow patients who previously did not respond to these drugs to have erections sufficient for penetration using them following LI-ESWT ^36^. Short-term outcomes are promising, but, as this is a new therapy, data regarding long-term outcomes are lacking. Kitrey *et al*. recently evaluated 156 patients who had undergone 12 treatment sessions over a nine-week period, with 5 treatment points along the penis and a total of 1,500 shockwaves administered at a frequency of 120 shocks per minute. These patients were followed for two years post-treatment. During the follow up period, the clinically effective response decreased over time, from 64% response rate one month following treatment to a 34% response rate at two years. This group hypothesized that a higher rate of treatment failure may be related to more-severe ED and more medical comorbidities at baseline prior to the start of treatment ^37^. Though preliminary results with LI-ESWT are promising, additional research needs to be done to determine long-term efficacy and side effects, and currently this treatment modality is offered only as part of a clinical trial.

### Stem cell therapy

There has been recent interest in the use of stem cells to treat ED. In animal studies, the injection of stem cells following prostate radiation has shown restoration of erectile function via the regeneration of cavernosal nerves ^38^. Small phase 1 studies in humans have shown promising results in terms of tolerability, safety, and efficacy of use of intracavernosal stem cell transplant for the treatment of ED ^39^. Interestingly, a recent study has suggested that the combination of LI-ESWT and stem cell therapy may promote the growth of new blood vessels and decrease the destruction of cells within the penile corpora, more than either therapy alone ^40^. Much more work needs to be done in this field to understand the long-term efficacy and safety of this therapy, which remains investigational at this time.

### Platelet-rich plasma

Platelets produce growth factors which are important in wound healing and the growth of new blood vessels. Intracavernosal injection of PRP in animal models has shown improvement in erectile function in cases of neurogenic ED ^41^. This has been translated into human studies, and a recent study of four patients with ED treated with PRP resulted in improvement in erectile function, with no serious adverse events ^42^. Additional studies need to be done to determine if this is reproducible on a larger scale.

### External penile prosthesis

Despite advances in medical and surgical treatments for ED, many patients may have an incomplete or unsatisfactory result to treatment or may not be able to afford more-invasive surgical treatments ^43^. Over-the-counter sexual aids may be a more affordable, minimally invasive, and effective alternative to traditional ED treatments. The challenge with the use of these devices may be twofold: physicians may be less knowledgeable about the variety and cost of these options, and patients may find the notion of sexual aids to be stigmatized with a vulgar or embarrassing connotation ^26^. It has been suggested that describing sexual aids as external penile prostheses may “medicalize” these tools and may increase the physician’s comfort in prescribing these devices and increase patient willingness to use them ^43, 44^.

A variety of external devices exist as sexual aids, including penile sleeves, extenders, support devices, and the banded prosthetic phallus ^26^. These devices allow for the preservation of penetrative intercourse in the setting of ED and may be combined with various physical shapes or vibratory stimulation technology to enhance intercourse.

The external penile prosthesis is a prosthetic phallus that is strapped around the patient’s waist. While it may seem difficult to conceive that a prosthetic phallus could result in rewarding penetrative intercourse for the patient, the neurophysiology surrounding the perception of appendages is complex, and it has been suggested that the incorporation of other senses can allow for a sensation that the prosthetic phallus is the patient’s own phallus, and orgasm may still be achieved by the patient and partner with the use of an external penile prosthesis ^26^.

Satisfaction with these treatment options depends on both the patient and the partner, so extensive counselling should be provided in the office to maximize success rates ^26^. Little research exists regarding the effectiveness and variety of external prosthetic devices and alternative treatments for ED, but these treatment options may be more accessible, less invasive, and more cost effective for the appropriate group of patients. More work needs to be done to understand patient and partner outcomes following treatment with these prosthetic devices.

## Conclusion

There have been many developments in treatments for ED. Traditional therapies including lifestyle modifications, oral medications, injections, and penile prostheses have shown great efficacy in the management of ED. However, research on novel approaches remains sparse, and alternative treatment modalities including external penile prostheses have been slow to gain acceptance in the scientific community. External penile prostheses and LI-ESWT have shown promise as treatment options for men with ED, with few side effects and high cost-effectiveness. More research needs to be done to fully appreciate the role these options may play in the management of ED.

## Abbreviations

AUA, American Urological Association; cGMP, cyclic guanosine monophosphate; ED, erectile dysfunction; GTP, guanosine triphosphate; ICI, intracavernosal injection; LI-ESWT, low-intensity extracorporeal shockwave therapy; NO, nitric oxide; PDE5, phosphodiesterase type 5; PGE1, prostaglandin E1; PRP, platelet-rich plasma; VED, vacuum-assisted erectile device.
